# A Huge Mesenteric Lymphangioma Presenting as a Small Bowel Volvulus in a Paediatric Patient: A Case Report

**DOI:** 10.1155/2022/3033705

**Published:** 2022-05-17

**Authors:** Sushma Thapa, Abhinav Sharma, Dipesh Upreti, Om Bahadur Karki, Sudeep Regmi, Dilasma Ghartimagar, Arnab Ghosh

**Affiliations:** ^1^Department of Pathology, Manipal College of Medical Sciences, Pokhara, Nepal; ^2^Department of Surgery, Adesh Medical College and Hospital, Ambala, Haryana, India; ^3^Department of Surgery, Manipal College of Medical Sciences, Pokhara, Nepal

## Abstract

Lymphangioma is a benign tumor characterized by proliferation of thin-walled lymphatic spaces. Lymphangioma of the small-bowel mesentery is rare, with an incidence of 1 : 250,000, representing less than 1% of all lymphangiomas. The predilection of the tumor is in the head and neck (70%), axillary (20%), and internal organs (10%). They are usually asymptomatic but can cause acute abdominal symptoms due to complications such as volvulus, bleeding, or lymphangioma rupture that require emergent surgery. Here, we report a case of mesenteric lymphangioma (ML) of a small bowel in a paediatric patient who presented with pain abdomen on and off which increased in severity and later had features of subacute intestinal obstruction. He underwent explorative laparotomy, and the mass was excised completely along with the part of small intestine. Pathological analysis of the surgical specimen confirmed the diagnosis of ML of the small intestine. The postoperative recovery was uneventful, and the patient was discharged after ten days of hospital stay. Though benign in nature, ML may cause acute abdominal symptoms that require emergent surgery. Therefore, it has to be kept in differential diagnosis of the acute abdominal condition.

## 1. Introduction

Lymphangioma is a benign tumor characterized by proliferation of thin-walled lymphatic spaces [1]. Lymphangioma of the small-bowel mesentery is rare, with an incidence of 1 : 250,000, representing less than 1% of all lymphangiomas [2, 3]. It is commonly found in children and represents 5-6% of benign tumors in children appearing 60% at birth and 40% by one year of age [4, 5]. The predilection of the tumor is in the head and neck (70%), axillary (20%), and internal organs (10%) [6]. The majorities of abdominal lymphangiomas, which account for 1% of all the lymphangioma cases, are commonly of cystic type and occur in the mesentery, followed by the omentum, mesocolon, and retroperitoneum [7]. They are usually asymptomatic but can cause acute abdominal symptoms that require emergent surgery [8]. Diagnosis is confirmed by histopathological findings of lymphatic vessels restricted to the connective tissue of endothelial cells and smooth muscle tissues [5].

Here, we report a case of a huge mesenteric lymphangioma of small intestine in a 3-year-old male child presenting with features of partial intestinal obstruction.

## 2. Case Report

A 3-year-old male child presented with pain in the abdomen on and off for 2 years with increased severity since 7 days. The pain was more during the night time and aggravated while feeding. It was associated with nonprojectile vomiting. There was also history of passage of hard stool once in 4-5 days, and since 2 days, the child had not passed urine. According to the father, the child had lost some weight. His past medical, surgical, and family history was unremarkable. On general examination, the child appeared malnourished and pale. On per abdominal examination, a mass of around 3 × 3 cm was palpable just below the umbilical region. There was no abdominal distension. Bowel sounds were present. Other systemic examinations were normal.

The laboratory investigations during the time of admission, viz., complete blood count, PT-INR, and serum electrolytes, were within normal limit. However, blood urea was 53.03 mmol/L (3.57-16.07 mmol/L), and serum creatinine was 176.84 *μ*mol/L. His random blood sugar was 4.44 mmol/L (4.55-7.77 mmol/L). Gradually, after few days of admission, the renal function test returned to normal limit. All serological tests for human immunodeficiency virus, hepatitis B virus, and hepatitis C virus were negative. The total protein was within normal limit, i.e., 45 gm/L (63-82 gm/L). His routine urine examination showed 2-3 white blood cells per high power field.

Ultrasonography (USG) of the abdomen showed an echogenic lesion measuring 5.5 × 5.1 cm in the lower abdomen and pelvis with twisting of vessels and mesentery in the periumbilical region. It was reported as differential diagnosis of mesenteric mass and thickened mesentry.

Computed tomography (CT) scan of the abdomen and pelvis revealed a large well-defined, lobulated homogenous nonenhancing cystic mass lesion with thin walled internal septation in the peritoneal cavity measuring 15.7 × 6.4 × 11.5 cm and was reported as mesenteric lymphangioma. There was also swirling appearance of the mesentery and superior mesenteric vein around the superior mesenteric artery in an anticlockwise direction representing midgut malrotation with middilatation of bowel loops suggesting volvulus.

**Figure 1:**
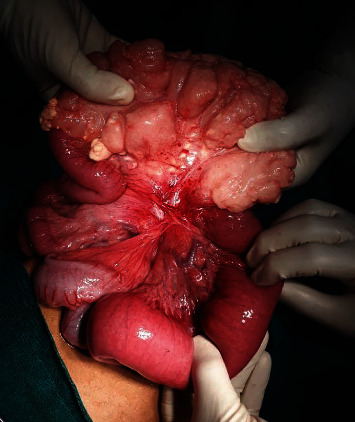
Intraoperative image showing a huge lobulated, multicystic mesenteric mass attached to the small intestine.

Laparotomy was performed. During the operation, a huge mesenteric tumor mass measuring 15 × 14 cm attached with the small intestine along with midgut malrotation was found. The tumor was excised completely with small bowel resection comprising of around 10 cm of distal jejunum and proximal ileum followed by anastomosis of proximal to distal segment (Figure 1). The operation procedure was uneventful with minimal blood loss (approximately 100 mL) and took only around 45 minutes to complete. The procedure was well tolerated by the patient without any clinically significant postoperative complications.

**Figure 2:**
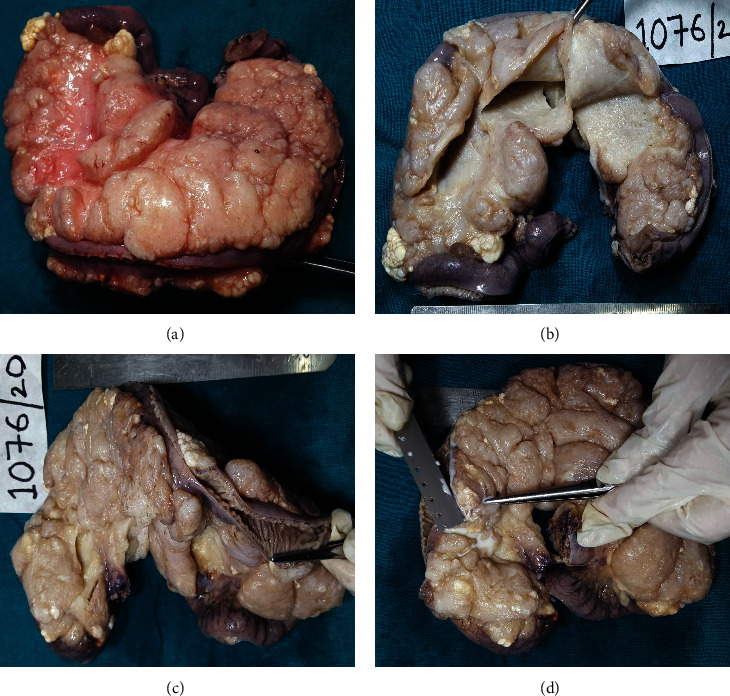
Gross photograph of mesenteric lymphangioma of small intestine: (a) postoperative specimen. (b) Formalin-fixed specimen with section through the mass showing pale white solid tan to spongy appearances, (c) involvement of small intestine, and (d) oozing of the milky fluid.

The tumor was received in the department of pathology as a huge lobulated, semitranslucent, pale white mass measuring 24 cm in maximum dimension with attached small intestine measuring 43 cm in total length. Externally, multiple areas showed chalky white nodular areas largest measuring 2 cm and smallest measuring 0.5 cm in maximum dimension. On serial sectioning, the cut surfaces showed multiple minute cystic cavities giving a spongy appearance and were filled with milky fluid (Figure 2(a)). On cut opening through the intestine, multiple focal nodular areas identified size ranging from 0.8 to 3 cm in maximum dimension with cut surface showing milky fluid. The rest of the mucosa was unremarkable (Figures 2(b)–2(d)).

**Figure 3:**
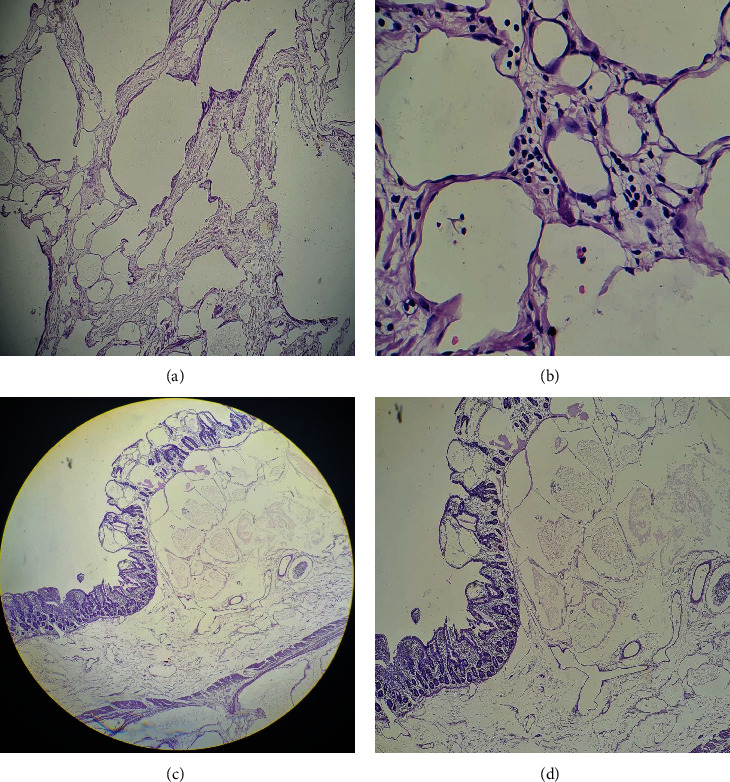
(a) Photomicrograph of mesenteric lymphangioma showing numerous cystically lymphatic channels (hematoxylin & eosin stain, 4x); (b) lined by flattened endothelium and perivascular lymphoid infiltration; (hematoxylin & eosin stain, 40x); (c, d) involvement of mucosa, submucosa, and muscularis propria by lymphangioma (hematoxylin & eosin, 4x and 10x).

Histologically, section from the main mass showed several cystically dilated, various calibered lymphatic channels lined by flattened endothelium. Some of the lumen showed eosinophilic secretions with some showing few collections of lymphocytes and occasional RBCs. The intervening thin fibrocollagenous stroma was edematous and shows sparse lymphocytic infiltrate and congested blood vessels. Section from the intestine also showed lymphatic channels involving the mucosa, submucosa, and muscularis propria (Figures 3(a)–3(d)). The final pathological analysis was lymphangioma small intestine with multiple focal areas of involvement of mucosa, submucosa, and muscularis propria. The patient was discharged on the tenth postoperative day. At one-year follow-up, he was in good health.

## 3. Discussion

Lymphangioma is a mass-forming lesion characterized by numerous thin-walled lymphatic spaces and usually manifests in the first few years of life, and the sex ratio is roughly equal during the childhood [1]. The common predilections are head, neck, and axillary regions. Other locations such as the abdominal or mediastinal cavity are rare, accounting for approximately 5% of lymphangiomas [2]. Among these, lymphangioma of the small-bowel mesentery are very rare, and lymphangiomas in the jejunum or ileum are extremely rare, accounting for less than 1% of all lymphangiomas [1, 9]. A literature review of studies on lymphangiomas revealed that only 19 cases of small bowel lymphangioma were reported from 1960 to 2009 [10].

Lymphangioma appears to result from congenital malformation of lymphatic vessels rather than a true lymphatic tumor [2, 11]. The former causes sequestration of lymphatic vessels during the embryonic period [1]. However, some data suggest that inflammation, abdominal trauma, abdominal surgery, radiation, or lymphatic obstruction may play a role in the genesis of a tumor [12, 13]. Notably, the patient described in the current case study had a history of intermittent pain abdomen without identifiable cause from the time of 1-year old. This supports the theory of lymphangioma resulting from congenital malformation of lymphatic vessels rather than a tumor.

Mesenteric lymphangioma (ML) may remain asymptomatic though the clinical presentation can range from an incidentally discovered abdominal mass to symptoms of an acute abdomen (volvulus or intestinal obstruction) depending on the size and location of the mass [3, 14, 15]. Acute intestinal obstruction in ML occurs in the form of small-bowel volvulus [11]. Small-bowel volvulus is the rotation of the small bowel and its mesentery and is complicated by acute intestinal obstruction. The precipitating factors to volvulus include postoperative adhesion bands, congenital bands, colostomy, ileostomy, fistula, tumors, omental defect, and Meckel's diverticulum [16]. ML induces rotation of the small bowel, resulting in small-bowel volvulus with subsequent closed-loop small-bowel obstruction as observed in the current study [9]. Although benign, lymphangioma can cause other symptoms such as bleeding, torsion, or lymphangioma rupture [17]. Therefore, ML should be considered as one of the differential diagnosis in the acute abdominal cases in children [5].

Classical imaging findings are those of a thin-walled multiloculated cystic lesion lacking solid components or mural nodularity. Calcifications may occur, but are uncommon [18]. The wall and septae are typically nonenhancing or minimally enhancing. Lymphangiomas typically show T2 high signal fluid, while signal loss may be visualized on chemical shift T1-weighted imaging due to microscopic fat content from chylous fluid which is a very suggestive finding for a lymphangioma occurring in 20-30% of cases, although rarely it may be found in other limited differentials such as lymphoceles, lymphoepithelial cysts, and pancreatic pseudocysts. Lymphangiomas may either be stable in size or show slow progressive growth. They typically show little in the way of mass effect and generally insinuate around adjacent structures. Lymphangiomas are not associated with local invasion, enlarged adenopathy, or organ metastases. The imaging algorithm for the work-up of lymphangiomas would depend on the local resources and availability of imaging modalities such as USG, CT, and magnetic resonance imaging (MRI) [19]. USG and CT are considered as the most appropriate radio-diagnostic modalities to evaluate ML, although ultrasonography is usually sufficient. USG also can differentiate the lymphangiomas from simple mesenteric cyst, because the fluid contents in lymphangiomas are usually homogeneous, and attenuation values may range between those of fat and fluid [8]. However in our cases, USG abdomen showed an echogenic lesion in the lower abdomen and pelvis with twisting of vessels and mesentery in the periumbilical region with differentials of mesenteric mass and thickened mesentry, and CT scan was advised. On CT scans, ML appeared as uni- or multilocular masses containing septa of variable thickness; enhancement of the wall is revealed by contrast medium similar to the present study [8].

The site and size of the lymphangiomas varies. One case report published in 2012 described a lesion measuring 5.0 cm × 4.0 cm in duodenum [20]. In the present case, the lesion was in the small intestine and measured 24 cm in maximum dimension. Lymphangioma are traditionally classified into three histologic types: capillary (simple), cavernous, and cystic [1]. The capillary (simple) type usually originates in the skin and consists of uniform small thin-walled lymphatic spaces. The cavernous type is composed of various sizes of dilated lymphatic spaces associated with lymphoid stroma and shows a connection with the adjacent normal lymphatic spaces. The cystic type consists of dilated lymphatic spaces of various sizes associated with collagen and smooth muscle bundles in the stroma but lacks connection to the adjacent normal lymphatic spaces. Cystic lymphangioma findings are similar to cavernous lymphangioma findings in that dilated lymphatic spaces of variable size are seen for both [21].

Immnunohistochemical studies like factor VIII-related antigen and D2-40 can be done to demonstrate lymphatic endothelial markers as a supportive findings for the diagnosis, but in our hospital, this set up was not present, so diagnosis was mainly based on the presence of milky white fluid content on the gross section supported by the presence of lymphoid follicles and lymphoid infiltrates in the stroma histologically [22, 23].

Mesenteric lymphangiomas are very rare, but they can cause acute abdominal symptoms that require emergent surgery. In addition, ML may also cause other complications such as infiltration of the intestine or involvement of the main branch of the mesenteric arteries or adjacent organs that necessitate segmental resection of the intestine [21, 24, 25]. Surgical segmental bowel resection, including the lesion, is the optimal treatment for avoiding recurrence [26]. In the present study, also, the patient underwent surgical resection of a small bowel segment including the lesion. If radical surgery is not technically possible, injection of bleomycin or OK-432 into the tumour has been reported to be effective by few authors [1].

## 4. Conclusion

Although benign in nature, ML may cause significant morbidity or mortality due to their large size, and critical location may compress the adjacent structures. It can cause acute abdominal symptoms that require emergent surgery. Therefore, it should be included in the differential diagnosis of the acute abdominal condition.
